# Tales of Tails

**DOI:** 10.3390/e25040598

**Published:** 2023-03-31

**Authors:** Christopher Essex, Bjarne Andresen

**Affiliations:** 1 Department of Mathematics, Middlesex College, The University of Western Ontario, London, ON N6A 5C1, Canada; essex@uwo.ca; 2 Niels Bohr Institute, University of Copenhagen, Blegdamsvej 17, DK-2100 Copenhagen Ø, Denmark

**Keywords:** probability density distributions, effect of tails, thermodynamics, very long timescales, slow time

## Abstract

Typical human-scaled considerations of thermodynamic states depend primarily on the core of associated speed or other relevant distributions, because the wings of those distributions are so improbable that they cannot contribute significantly to averages. However, for long timescale regimes (slow time), previous papers have shown otherwise. Fluctuating local equilibrium systems have been proven to have distributions with non-Gaussian tails demanding more careful treatment. That has not been needed in traditional statistical mechanics. The resulting non-Gaussian distributions do not admit notions such as temperature; that is, a global temperature is not defined even if local regimes have meaningful temperatures. A fluctuating local thermodynamic equilibrium implies that any local detector is exposed to sequences of local states which collectively induce the non-Gaussian forms. This paper shows why tail behavior is observationally challenging, how the convolutions that produce non-Gaussian behavior are directly linked to time-coarse graining, how a fluctuating local equilibrium system does not need to have a collective temperature, and how truncating the tails in the convolution probability density function (PDF) produces even more non-Gaussian behaviors.

## 1. Introduction

The well-known pitch drop experiment [1] concerns extremely long-time behavior in highly viscous pitch. The hanging pitch seems to be fixed and unmoving. However, droplets have formed and fallen nine times since 1927. No one has ever witnessed a fall.

This experiment illustrates the issue. Some processes are too slow to observe (the extremely slow drop formation, if one were to sit and wait for it), while others are too fast to observe (the drop fall, if one were to check on the experiment only daily) [2]. Such rare events, as well as those too slow to see, are normal parts of our world but are too easily overlooked. Nonetheless, the outcome of drops on our timescales illustrates one way that slow processes can become important. Our work has focused on such processes on ultra-long timescales. This has been dubbed “slow time”.

This was inspired originally by time exposure photographs of the Niagara River [3]. In such pictures, the existence of slow processes invisible to the eye is revealed through the time coarsening intrinsic to time exposures. Such coarsening is a model for how to think of transitions between different time/space regimes [4]. Just like some features disappear and some new ones emerge in the transition from molecular time/space scales to a human scale, we expect (and find) similar disappearance and emergence when employing the even slower and larger slow time regime. New fundamental thermodynamics is uncovered in the process.

The time exposures of turbulent media, such as the Niagara River, and of local equilibrium fluctuations, such as those one encounters from weather [5], have a natural mathematical analogue. That is the evolution of probability density functions (PDFs) as one encounters sequences of fluctuating local states over time. In practice, this involves fluctuating local state variables, such as wind speed and temperature, to achieve a representation. These fluctuations amount in practice to convolving local Maxwellian PDFs with PDFs for the fluctuating variables. Such fluctuations are often not of the same simple type as those encountered with molecular fluctuations in a fluid in equilibrium (Brownian motion). A direct connection from convolution to time coarsening is made in Section 3. Related concerns appeared 20 years ago but did not take hold [6].

In our work on slow time, it has become evident that transitions from one time/space realm to another crucially depend on the tails of the probability density functions involved. For example, in [2] we coarse-grained the wind speed and temperature over large times/areas, assuming a Gaussian distribution of the corresponding Gaussian precisions (inverses of standard deviations divided by $\sqrt{2}$) at the smaller scale. We found that wind may be coarse-grained, leading to an appropriate adjustment of the temperature to include the wind fluctuations on that longer timescale (thermalizing the wind in effect). Think of Brownian motion on an unusually long timescale. This results in a new temperature with higher values that amount to the “thermalization” of wind.

Temperature itself, on the other hand, could not be thermalized in the same way because the ensuing PDF was not of Maxwellian (thermal) type and thus did not allow for a temperature. Here and in the following, our arguments concern a classical ideal gas (see, for example, [7]) such that the usual definition of temperature through the equation $1/T={\left(\partial S/\partial U\right)}_{N}$ is equivalent to the Maxwellian velocity distribution $p\left(v\right)=\sqrt{m/2\pi kT}\;\exp\left(-mv^{2}/2kT\right)$. The result was a completely unprecedented PDF form: Maxwellian in the core with heavy polynomial tails. In addition to not being consistent with the existence of a temperature, the non-Gaussian heavy tails (i.e., the approach to zero as powers rather than exponentially) resulted in some divergent standard moments needed for macroscopic mathematical relationships. This means that the fluctuating local equilibrium at the root of such convolutions does not allow for an “averaged” temperature. To reach an understanding of the impossibility of such a temperature in this context, think of a system consisting of 10 kg of ice at −5 °C and 1 kg of water at 50 °C. Does that system have an average temperature, and if so, what is it?

Many unusual questions emerged. Why do heavy tails develop when there are none for Maxwellian PDFs? How do we cope with divergent integrals if one seeks the moments of the new PDF? If there is no temperature, then how can there be entropy and energy across a fluctuating local equilibrium field without a function to relate them to each other? This paper addresses such questions.

The primary source of these questions is a lack of experience with the PDF tails arising from the physics. Section 2 addresses why tails in PDFs do not easily appear in direct observations. For certain questions, they are simply insignificant should they actually show up. However, when addressing slow time, they emerge as important nonetheless.

Section 3 shows how a general process of PDF evolution representing time coarsening works, providing a direct link to convolution. Non-Gaussian tails, implying no temperature, indicate the absence of equilibrium and hence the non-existence of a function of state. However, in the local equilibrium picture, the local extensities (e.g., entropy and energy) can be summed due to their essential additivity to gain global values. At the same time, the non-Gaussian tails also imply that there are no global intensities, as the overall system is out of equilibrium. This means that there is no function of state globally, even though there is one locally.

Section 4 builds on a previous example [3] on closure to show how a local function does not imply the existence of a global one. We accomplish this by showing how entire regions of points may arise from more than one class of underlying functions. This is induced by the fluctuating structure of the essential underlying scalar fields arising from the fluctuating local equilibrium. Those fields are fundamentally dynamical and independent of the local thermodynamics.

Finally, Section 5 uses a convolution PDF with suppressed tails (a box car) to see the effect that this might have on treating the problem of divergent integrals. It turns out that this fix to the problem leads to sub-Gaussian tails, but it retains a Gaussian core. Despite being sub-Gaussian, this remains curiously similar to previous long-time PDFs, as the core is Gaussian while the tails are not. Thus, there is still no formal temperature.

## 2. Problems with Observing Known PDF Tails Directly

Previously [2], we had to contend with the divergence of integral moments appearing in quantities such as internal energy. Such divergences are ultimately not physical, as the PDFs must be false for large values of speed, if for no other reason that the distribution cannot include speeds that exceed the speed of light. Such a limitation is understandably not considered for the Maxwellian PDF where all moments converge. Depending on the details, this relativistic limit may be beyond another one wherein all of the kinetic energy in a collection of particles goes to only one of the particles [2]. Beyond that limit, the real physical distribution must be fully depopulated.

Thus, the true PDF can only be non-zero over a finite domain. In practice, the transition to full depopulation will likely take place over an interval in velocity space and not happen at one value. Over that transition interval, between being fully populated and empty, any continuum representation of the PDF will not be valid. The question arises as to how easy that might be to detect directly, let alone to even observe the far tails of ideal known PDFs directly.

Setting aside possible indirect observation, let us look at how one might measure a known ideal PDF directly. Assume that we have an ideal gas in a standard thermal Gaussian molecular speed distribution centered around *u*:

(1) $$\begin{array}{c} p_{u}\left(v\right)={\left(\frac{m}{2kT}\right)}^{1/2}\frac{1}{\sqrt{\pi }}\;e^{-\frac{m}{2kT}{\left(v-u\right)}^{2}}, \end{array}$$

where *m* is the particle mass, *k* the Boltzmann constant, and *T* the temperature. If we set u=0, then

(2) $$\begin{array}{c} p\left(v\right)={\left(\frac{m}{2kT}\right)}^{1/2}\frac{1}{\sqrt{\pi }}\;e^{-\frac{m}{2kT}v^{2}}=\frac{1}{\sigma \sqrt{2\pi }}\;e^{-\frac{v^{2}}{2\sigma^{2}}}, \end{array}$$

where σ is the standard deviation.

**Figure 1:**
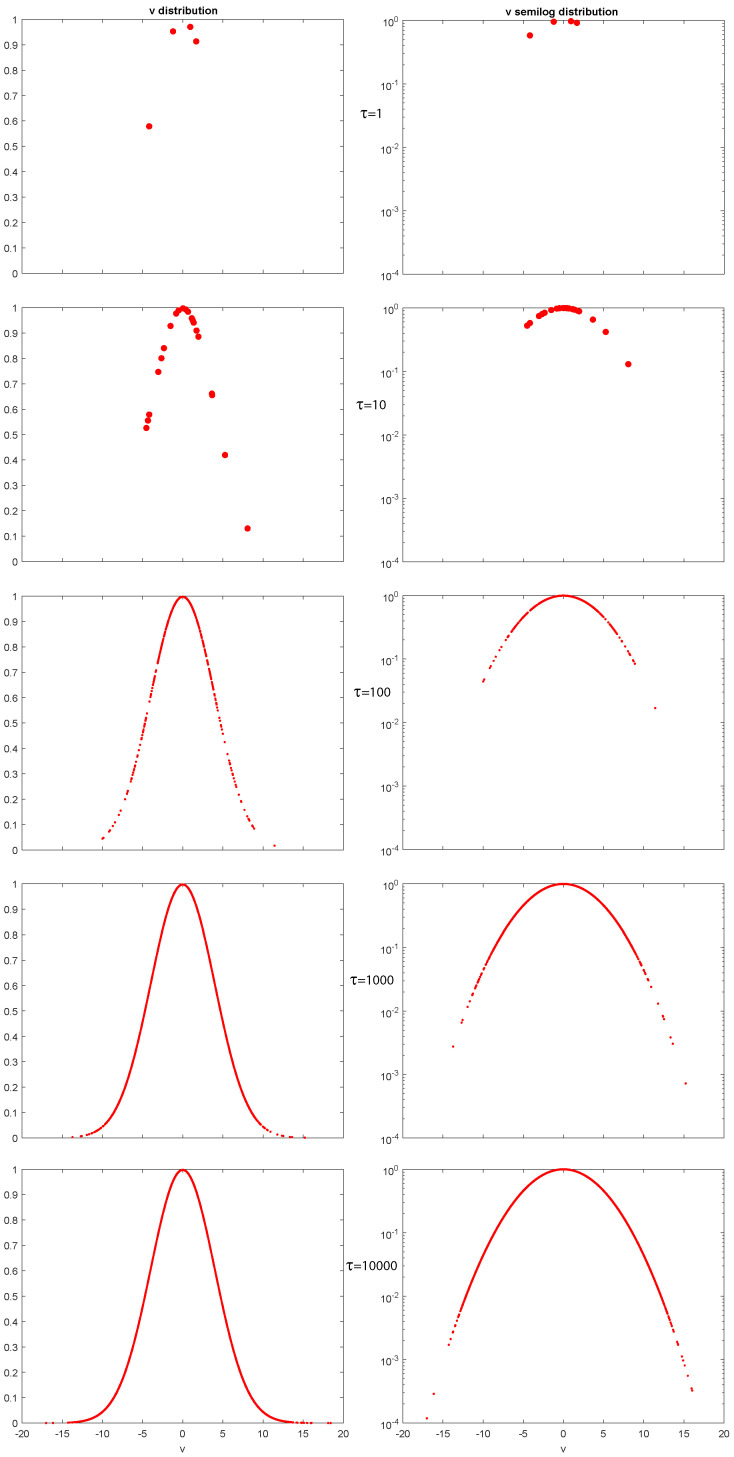
Sequence of samplings of a Gaussian distribution with standard deviation σ=4. The top row samples it for a period τ, while the subsequent rows are for periods 10,100,1000, and 10,000 times longer, respectively. The left column contains linear plots, while the right column is on a semi-log scale. Being of exponentially small probability, the wings only begin to be observed for long sampling times and even then are never entirely seen.

In order to observe this distribution, we need to count the particles within given speed intervals. Figure 1 shows such a sequence of measurements where, for illustrative purposes, we set σ=4, and the graph was scaled to require the peak value to be one. The top row simulates the counts by a detector covering the velocity range [−20,20] from a total of 10 particles impinging. Here, four counts were recorded in this run. Another realization might detect more or less of the 10 particles.

Suppose it took a measurement time interval τ for the 10 particles to pass or hit the detector. In addition, suppose that the flow of particles to the detector was steady. Then, the next rows represent the same detector recording counts over time intervals of $10\;\tau ,100\;\tau ,1000\;\tau ,10^{4}\;\tau$, and $10^{5}\;\tau$. The more patricles that are detected, the better a picture we obtain of the distribution, which of course is always predominantly around v=0, as these are simply the most probable values.

The outcomes are plotted on a linear scale in the left column. By the time the bottom row is achieved, the Gaussian function seems to be fairly complete. The sequence is most reminiscent of the build-up of images during a photographic time exposure. Initially, with low photon counts, the image is barely discernible, building to a full picture when the exposure ends. One can literally see the picture emerge gradually as time passes and more and more photons arrive.

The semi-log plots in the right column show a different perspective. The data quickly fills up at the center of the distribution in both the linear plot and the semi-log plot. However, as one moves away from the center, the semi-log case always becomes progressively more sparse (and eventually empty) in the tails. Even in the most numerous case in the bottom row, the tails are empty. Each factor of 10 in time only advances the semi-log curve linearly, always leaving depopulated space representing the tails below.

For our example, it is essential to notice that the observer never obtains the full probability distribution. The far wings have such low probabilities that the chance of observing a count there is essentially zero. The tails become all but invisible in the case of direct observation. Thus, it is understandable that they prove immaterial for many practical questions. However, while the tails may seem to be immaterial on our usual laboratory timescale, for long timescales (slow time), we have found that the tails play a significant role after all.

Although integrals will not be divergent in the real everyday world even if the PDF is not truly Gaussian, this does not imply that all usual thermodynamic variables must therefore exist across regimes. For example, if the core (and thus detectable) part of the distribution is not Gaussian, then the concept of temperature will not carry over, regardless of the behavior of the far wings.

## 3. Fluctating Local Thermodynamic Equilibrium and Time

In this section, we leave behind the notion of incomplete PDF tails due to limits on the direct observation time and turn instead to the effects of the system going through a sequence of local states, which are transient. These fluctuations in the observed states are captured by a convolution with a new PDF that represents the fluctuations in speed and temperature. This procedure ultimately produces the non-Gaussian tails discussed previously [2]. While such a procedure seems intuitively equivalent to some kind of time coarsening, in this section, time coarsening is directly connected to convolution.

Let an initial probability density function (PDF) $P_{0}\left(V\right)$ be defined in terms of *n* variables *V* in a domain *W* such that $V\in W\subseteq R^{n}$. Then, $P_{0}\left(V\right)\geq 0$ and $\int_{W}P_{0}\left(V\right)dV=1.$ A sequence of PDFs, $P_{i}\left(V\right)$, where i=0,1,2…, is induced by a map

(3) $$\begin{array}{c} P_{i}\left(V\right)\to P_{i+1}\left(V\right) \end{array}$$

which is defined by

(4) $$\begin{array}{c} P_{i}\left(V\right)+R_{i+1}\left(V\right)=P_{i+1}\left(V\right). \end{array}$$

Thus, $R_{i+1}\left(V\right)$ represents how much the measured probability density function $P_{i}\left(V\right)$ changes to arrive at the next PDF, $P_{i+1}\left(V\right)$, and thus $R_{i}\left(V\right)$ is one of a sequence of *redistribution functions* [8]. They have the property

(5) $$\begin{array}{c} \int_{W}R_{i}\left(V\right)dV=0. \end{array}$$

The special case where $\int_{W}\left|R_{i}^{0}\left(V\right)\right|dV=0$ implies $R_{i}^{0}\left(V\right)\equiv 0$, which we will call the null redistribution. It defines the identity operation of the map, but in the fluctuating local equilibrium environment envisaged, it will not play a significant role.

The redistribution functions are malleable in the sense that $\sum_{k=j}^{m}R_{k}\left(V\right)$ is also a redistribution function. Thus, any sequence of maps can be reduced to a single map step,

(6) $$\begin{array}{c} P_{j-1}\left(V\right)+\sum_{k=j}^{m}R_{k}\left(V\right)=P_{m}\left(V\right) \end{array}$$

as a result of the simple additivity of the map. The sum can be replaced by a new redistribution function $R_{m}$ before adjusting the subscripts appropriately. In redefining the counting index to convey a single step to reach $P_{m}$, we return to Equation (4).

Each PDF and redistribution function is equipped with a time resolution; that is, any detector takes time to accumulate enough data to establish some particular observed function. However, setting that aside, from a thermodynamics point of view, in a fluctuating local equilibrium, the detector is exposed to a sequence of local equilibrium states accounting for the redistributions in play. There is a timescale for each exposure contributing to the effective timescale of each redistribution of the PDF; this is time coarsening. For the map, this implies a cumulative coarsening with each iteration. For example, we have

(7) $$\begin{array}{c} \underset{\Delta t_{0}}{\underset{︸}{P_{0}\left(V\right)}}+\underset{\Delta t_{1}}{\underset{︸}{R_{1}\left(V\right)}}=\underset{\Delta t_{0}+\Delta t_{1}}{\underset{︸}{P_{1}\left(V\right)}}. \end{array}$$

The Δts represent the intervals over which events cannot be resolved or cannot be ordered in time, much as found in the photographic time exposures. Just, the exposures are about how long the detector experiences the individual states as they pass the detector. We observe that the time coarsening is cumulative. The time resolution of $P_{1}$ is $\Delta t_{0}+\Delta t_{1}$, which we will call $\tau_{1}$. Extending this concept, we may generally write

(8) $$\begin{array}{c} P_{0}\left(V\right)+R_{1}\left(V\right)+R_{2}\left(V\right)+\cdots +R_{m-1}\left(V\right)+R_{m}\left(V\right)=\underset{\tau_{m}}{\underset{︸}{P_{m}\left(V\right)}}. \end{array}$$

In the environment of sequential fluctuating states, $P_{m}\left(V\right)$ may act as a nearly stable PDF, or it could be a transitional PDF. There is no requirement that the series converges, as there are only m+1 terms, representing the sequence of individual local states that pass by. This construction can be extended, of course, for an infinite series. If the infinite series does converge, then it would suggest a limiting PDF. Some evolution processes might not converge in the infinite case reflecting nonstationarity. Imagine, for example, a slow-time Brownian-like motion that can be observed on our human timescale but is invisible (smeared out) in slow time. This picture may provide insight with intermediate, transient PDFs as well as PDFs that are stable for practical purposes. If the latter is true, then suppose that the sequence of states is induced by some fluctuating parameter γ with domain *H* such that $P_{m}=P_{m}\left(V;\gamma \right)$. Here, γ might be, for example, wind speed or temperature. In other words, γ belongs to the human timescale but is invisible in slow time. We can then define a PDF, C(γ) distributed in γ, emerging from successive local states such that

(9) $$\begin{array}{c} P_{m}\left(V;\gamma \right)=\int_{H}P_{0}\left(V;\gamma \right)C\left(\gamma \right)d\gamma . \end{array}$$

Equation (9) is a convolution of $P_{0}$ and *C* of the type used in [2,8]. It follows that the convolution inherits the coarsened timescale $\tau_{m}$ from $P_{m}\left(V\right)$, thus demonstrating that a convolution with *C* directly implies a coarsened timescale $\tau_{m}$. In addition to time coarsening, the properties for the redistribution function series are more than broad enough to admit final PDFs that are in non-Gaussian forms.

## 4. No Temperature, No State Function, No Equilibrium

Mathematically speaking, thermodynamics in its classical form concerns functions, in contrast to dynamics, which concerns differential equations. For a fluctuating local thermodynamic equilibrium system (e.g., weather [5]), previous work has shown that a fluctuating local equilibrium system may have no global temperature [9]. As temperature is a slope on an equilibrium manifold (or “surface” in the case of the three extensities in the equation of state), no temperature implies that there can be no function. How can this be if there are thermodynamic functions that hold locally, as the concept of local thermodynamic equilibrium requires?

This section demonstrates that even though a local function may hold, there is still no need for a global function to exist, thus establishing that temperature can exist locally while not existing globally, as suggested by the non-Gaussian PDF tails. To accomplish this, we build on an example used previously. In [3], we dealt with the question of closure as a property of averaged systems. The simple illustrative example concerning functions at the lower scale not implying the existence of a function of the averaged variables was employed to make this point. We used the nonexistence of a function on the average to conclude that the averaged domain fails to have a relationship that is free of the need to refer to the underlying unaveraged regime. The averaged regime is not closed. There is no new regime, or one might say that the two realms exist while entangled.

In this section, the thermodynamic question is similar, but it requires some elaboration. Extensities such as energy and entropy may have global meaning physically, even in the absence of a global equilibrium function relating them. This is possible even if there exist *local* functions valid everywhere simultaneously that do relate them through thermodynamic functions. In contrast, intensities, such as temperature and pressure, may only exist locally and not globally. In [2], a physical PDF emerged that could not produce a temperature because of the fluctuating local equilibrium of the system, resulting in a non-Gaussian PDF (heavy tails). How can a global relationship fail to exist in this case?

We build on the preceding example [3] to demonstrate the nature of this failure in terms of simple functions, showing mathematical non-existence. We suppose that two one-parameter families of functions $\left\{g_{1}\left(s\right)\right\}=\left\{s^{d}|d>-1/2\right\}$ and $\{g_{2}\left(s\right)\}=\left\{s+b\right\}$ in parameters *b* and *d*, respectively, represent the evolving system on the fast (human) scale. One can think of these as, for example, entropy and energy and the variable *s* as a time or space variable on that same scale. The inequality on *d* prevents a singular result in integration, which becomes apparent below. $g_{1}\left(s\right)$ is a power relationship with the power *d* as a parameter, and $g_{2}\left(s\right)$ is a translation by the parameter *b*. Both are simple relationships, but they do not commute.

The objective here is to capture how local thermodynamic variables change over space. As the function shapes for such scalar fields vary widely, one may choose families of functions on an ad hoc basis for illustration without implying that these choices are found in nature or represent nature in any comprehensive manner. The only goal is to show that even with simple choices, functions fail to exist.

Next, we select an *ad hoc* average, which is chosen for simplicity rather than any attempt to claim how an average should be found. Such a simple average is

(10) $$\begin{array}{c} a_{h}=A\left\{h\left(s\right)\right\}\equiv \int_{0}^{1}h\left(s\right)ds, \end{array}$$

which is a function of *h*. This will bring us from the fine-grained scale to the coarse-grained slow time regime.

Then, we consider two functions, x(s) and y(s), where x(s) is either an element of the family $g_{1}\left(s\right)$ or an element of the family $g_{2}\left(s\right)$. We assume a simple relationship between the functions x(s) and y(s) that holds everywhere locally (i.e., on the fine-grained scale):

(11) $$\begin{array}{c} y\left(s\right)=x{\left(s\right)}^{2}. \end{array}$$

This fixed relationship, which is always locally valid, is the analogue for an equation of state. The families of functions $g_{1}$ and $g_{2}$ represent the local conditions as mentioned earlier and are the shapes of the local fields or intensities provided by processes such as “weather” (i.e., local temperature, pressure, humidity, etc.) in the configuration space. Time comes in through changes in the family of functions, i.e., the parameters *d* and *b*. One can imagine many more families of functions, but two are sufficient for our purpose.

If no free parameters are defined, then the averages *a* of the functions *x* and *y* will simply provide a single point in the $a_{x}$–$a_{y}$ space. That is not a function. With one parameter (*d* for $g_{1}$ and *b* for $g_{2}$), each class of functions produces a single averaged function, as we saw in [3]. This is achieved by simply solving for $a_{y}$ in terms of $a_{x}$ and, in doing so, eliminating the parameters. If x(s) is drawn only from the first family (i.e., $x=g_{1}\left(s\right)$) then this procedure yields

(12) $$\begin{array}{c} a_{y}=\frac{a_{x}}{2-a_{x}}\;\;;\;0\leq a_{x}<2 \end{array}$$

which is singular at $a_{x}=2$. If, correspondingly, x(s) is drawn only from the second family (i.e., $x=g_{2}\left(s\right)$), then

(13) $$\begin{array}{c} a_{y}=a_{x}^{2}+\frac{1}{12}. \end{array}$$

Note that $a_{x}=2$ in the $g_{1}$ case corresponds to d=−1/2, as noted above.

In both scenarios, after the single free parameter is eliminated, the averaged variables are indeed related by a function. However, if *both* classes are in play, then one cannot write $a_{y}=F\left(a_{x}\right)$, as *F* has more than one value at each $a_{x}$, such as when $a_{x}=0$, $a_{y}=1/12$ and 1/2. No *F* exists, except at the three values of $a_{x}$ where the curves of Equations (12) and (13) cross for $a_{x}=\left(\frac{1}{2},\;\frac{3}{4}+\frac{1}{4}\sqrt{\frac{11}{3}},\;\frac{3}{4}-\frac{1}{4}\sqrt{\frac{11}{3}}\right)$.

Do not imagine that this is merely an example of a “multi-valued” function for which “branches” may be selected. The two functions arising are unique only because of the deliberate simplicity of the chosen function classes and the limitation on the number of classes. In a randomly fluctuating local equilibrium field, the underlying functions would come from a much wider class, encompassing $g_{1}$ and $g_{2}$ as merely special cases.

To illustrate this, we can expand the two function classes by introducing more parameters. For example, let $\left\{g_{1}\left(s\right)\right\}=\left\{cs^{d}|d>-1/2\right\}$ and $\{g_{2}\left(s\right)\}=\left\{ks+b\right\}$. Then, for $g_{1}$, if we hold *c* at a fixed value and eliminate *d*, then we find

(14) $$\begin{array}{c} a_{y}=\frac{c^{2}a_{x}}{2c-a_{x}}\;\;;\;0\leq a_{x}<2c\;. \end{array}$$

Similarly, for $g_{2}$, when holding *k* constant, we find

(15) $$\begin{array}{c} a_{y}=a_{x}^{2}+\frac{k^{2}}{12}\;. \end{array}$$

**Figure 2:**
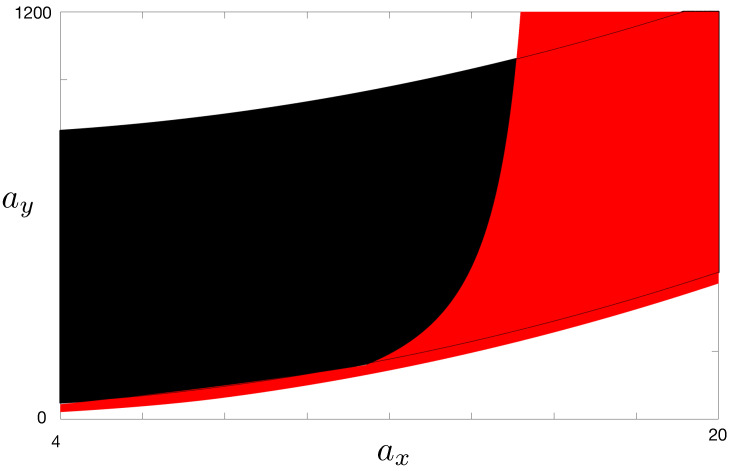
Suitable ranges of *k* and *c* in the relations in Equations (14) and (15) generate space-filling areas in this $a_{x}$–$a_{y}$ plot, indicating that no function exists relating $a_{x}$ to $a_{y}$, since both function families $g_{1}$ and $g_{2}$ are in play. No function exists, even though a relationship always exists between *y* and *x* in Equation (11). The $g_{2}$ family is shown in black, while the $g_{1}$ family is in red. The two areas overlap in the right half of the figure, indicated by the thin black lines. The boundaries of regions are envelopes made up of segments from potentially more than one function family member.

Neither of these are simple functions; rather, they are one-parameter families of functions filling space within chosen parameter domains. Given due consideration to the limiting domains, $a_{x}$ in either case produces infinite space-filling families of curves. What is more, these families overlap, making continuous domains of points spanned by either $g_{1}$ or $g_{2}$. In Figure 2, one sees $a_{y}$ vs. $a_{x}$. The black domain represents the $g_{2}$ family for 20<k<100, while the red domain represents the $g_{1}$ family for 8<c<20. Two separate black lines on the red domain denote the perimeter where the black domain is overlapped by red. These ranges of parameters are merely employed to produce an understandable image. The ranges could be expanded enough to make the resulting image difficult to interpret.

Functions map onto one and only one value. If there is more than one value, then the mapping is not a function, although the notion of a multi-valued function is a bastardization which is sometimes employed. However, continuous families of overwritten functions do not even meet the standard of the bastardization if there are no longer branches of $a_{y}=F\left(a_{x}\right)$. While $a_{y}$ and $a_{x}$ are well defined, there exists no function relating them. Thus, the slow time results in [2], wherein temperature fails to exist because of heavy tails, are not physically problematic. However, they do raise concerns about existing physical models for slowly varying noisy processes such as climate and weather [9,10,11].

## 5. PDFs on Finite Intervals

If tails are so important, then what if we truncate the tails in the convolution function? A “boxcar” PDF is used to address this. Consider a convolution function C=f(ξ) such that

(16) $$\begin{array}{c} f\left(\xi \right)=\left\{\begin{array}{c} 0\;\mathrm{for}\;|\xi |>\frac{1}{\omega }\\ \frac{\omega }{2}\;\mathrm{for}\;\left|\xi \right|\leq \frac{1}{\omega }, \end{array}\right. \end{array}$$

where *f* has an area of one and is a PDF which is known as a *box car* function. This serves as a departure from the Gaussian convolution functions employed in previous work on this topic. In particular, it is an example where the PDF tails are truncated. Such PDFs will arise from PDFs derived from any attracting set with a finite set diameter in a dynamical system (e.g., Lorenz equations).

We will convolve this with the PDF from [2]:

(17) $$\begin{array}{c} p\left(v\right)=\frac{\psi_{0}+\xi }{\sqrt{\pi }}\;e^{-{\left(\psi_{0}+\xi \right)}^{2}v^{2}} \end{array}$$

which arose previously after thermalizing wind. Here ψ is the Gaussian precision for temperature (precision is the reciprocal of the standard deviation except for a $\sqrt{2}$: $\psi =1/\sqrt{2}\sigma$), and ξ is the fluctuations about $\psi_{0}$ as the local equilibrium states change, i.e., $\psi =\psi_{0}+\xi$; it is the internal parameter taking on the role of γ from Section 3.

In [2] we proved that temperature could not exist because the convolved temperature fluctuations produced a PDF with heavy tails. A Gaussian convolution function *C* led to the non-Gaussian form

(18) $$\begin{array}{c} p_{\mathrm{fat}}\left(v\right)=\frac{w^{3}\psi_{0}}{\sqrt{\pi }{\left(v^{2}+w^{2}\right)}^{3/2}}\;\exp\left(-\frac{w^{2}\psi_{0}^{2}v^{2}}{v^{2}+w^{2}}\right). \end{array}$$

Here, we use the box car function C=f(ξ) instead:

(19) $$\begin{array}{c} p_{\mathrm{slim}}\left(v\right)=\int_{-\frac{1}{\omega }}^{\frac{1}{\omega }}\frac{\psi_{0}+\xi }{\sqrt{\pi }}\;e^{-{\left(\psi_{0}+\xi \right)}^{2}v^{2}}\;\frac{\omega }{2}\;d\xi . \end{array}$$

The integral is easily evaluated to

(20) $$\begin{array}{c} p_{\mathrm{slim}}\left(v\right)=\frac{\omega }{4\sqrt{\pi }}\frac{1}{v^{2}}\left[1-e^{-\frac{4\psi_{0}}{\omega }v^{2}}\right]\;e^{-{\left(\psi_{0}-\frac{1}{\omega }\right)}^{2}v^{2}}. \end{array}$$

This is clearly non-Gaussian due to the pre-exponential factors. For small *v* values, we have

(21) $$\begin{array}{c} p_{\mathrm{slim}}\left(v\right)∼\;\frac{\psi_{0}}{\sqrt{\pi }}\;e^{-{\left(\psi_{0}-\frac{1}{\omega }\right)}^{2}v^{2}} \end{array}$$

and thus the Gaussian form survives but is not normalized in form. For small *v* values, $p_{\mathrm{slim}}\left(v\right)$ will be larger than a normalized Gaussian with its pre-factor of $\psi_{0}-1/\omega$ instead of $\psi_{0}$ for $p_{\mathrm{slim}}\left(v\right)$ above.

For large *v* values we have

(22) $$\begin{array}{c} p_{\mathrm{slim}}\left(v\right)∼\frac{\omega }{4\sqrt{\pi }}\frac{e^{-{\left(\psi_{0}-\frac{1}{\omega }\right)}^{2}v^{2}}}{v^{2}} \end{array}$$

which is also non-Gaussian. In fact, it is sub-Gaussian because of the factor of $1/v^{2}$.

This result is quite different from the asymptotic PDFs from [2],

(23) $$\begin{array}{c} p_{\mathrm{fat}}\left(v\right)∼\frac{w^{3}\psi_{0}}{\sqrt{\pi }}\;e^{-w^{2}\psi_{0}^{2}}\;\frac{1}{v^{3}} \end{array}$$

because its asymptotic form is polynomial and not exponential in *v*. Actually, the exponential does not contain *v* at all. In both cases, the core is Gaussian, but the tails are not. There is also no clear role for ω as the natural dividing value between large and small *v* values, unlike *w* in the previous calculations [2].

**Figure 3:**
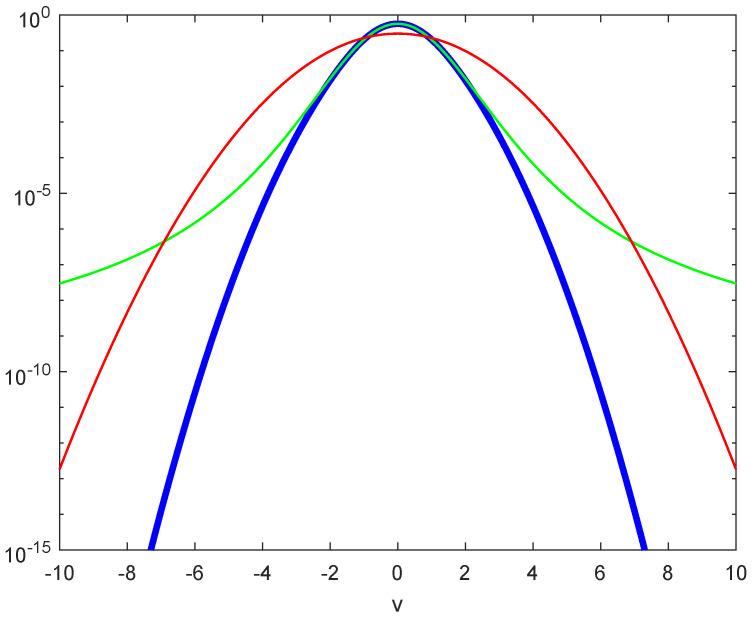
The fat tail speed distribution in Equation (18) arising from a convolution with Gaussian varying precision is shown in green. The blue curve represents a similar convolution but with a box car PDF, resulting in a sub-Gaussian speed distribution (Equation (20)). A standard Gaussian speed distribution (Equation (2)) is included in red for comparison. Not discernible in this logarithmic plot, emphasizing the tails, is that the green curve descends more rapidly than the blue curve around v=0, both starting from the same value of v=0. The green curve then crosses the blue one around v=±2, allowing a common normalization of 1 for all the distributions.

Figure 3 illustrates this. It is a semi-log plot which emphasizes the tail behaviors. We take the Gaussian (red) speed distribution as our reference point. Fluctuating the standard deviation in the basic speed distribution yields the fat tails of Equation (18) (green) and clearly shows a substantially larger probability for large speeds than the Gaussian, actually polynomial. The blue curve represents the slim box car convolution with ω=4. It is amazingly similar to $p_{\mathrm{fat}}\left(v\right)$ for small speeds and it decreases faster than the Gaussian curve for large *v* values, as indicated in Equation (22). Both convoluted curves rise above the Gaussian at the origin due to normalization.

The above analysis shows how sensitive a distribution convoluted with fluctuations really is. The central most populated parts of the convoluted distributions are hardly distinguishable to the naked eye, but the large deviation behaviors (the tails), even if the values are tiny, make all the difference for integrability and for identification with our conventional definitions of intensive thermodynamic quantities (e.g., temperature). Previously, one had to consider depopulation of the tails for physical reasons to make integrals converge. More exploration is needed for this topic.

## 6. Conclusions

This paper addresses a variety of questions arising from the surprising tail behaviors of PDFs designed to represent very long timescales. In the first instance, it is shown here, by a simulation, how direct observation of tails is challenging because of the improbability of observing events in tails. In practice, any direct observation of a PDF will have no measurement points beyond some finite distance from its center, no matter how much data is collected. Moreover, the time to fill each successive empty interval increases geometrically.

Previously [2], the accumulated distribution from a fluctuating local Maxwellian field was shown to have heavy tails, but a Maxwellian core as well. This unusual result was found by convolving Gaussian fluctuations of wind speed and temperature. The present paper elaborates on this observation and shows by means of redistribution functions [8] that such convolutions can be connected generally and directly to a timescale over which the temporal resolution is lost (i.e., time coarsening).

Given non-Gaussian tails, the classical conditions for generating temperature from statistical mechanical calculations do not hold. If they do not hold, can this notion agree with the idea that no state function can exist globally in a fluctuating local thermodynamic system? The present paper shows that local relationships do not imply global ones; that is, even if a local equation of state holds everywhere, there need not be one globally, which allows for a result with no global temperature for a non-Gaussian PDF.

If tails are the key to long-time behavior, is there a convolution PDF that can focus on this issue? To answer that question, we adopt a convolution function without tails: the box car distribution. This produces a surprising result. The core proves to be Gaussian as previously, but the tails are non-Gaussian in a new manner: They are sub-Gaussian! This also implies the absence of temperature. Instead, it suggests the possibility of a convolution function that has a Gaussian core and perhaps special tails such that a global temperature can exist. This unexpected result will figure into future work. 

## Data Availability

Not applicable.
